# Amyloid Proteins and Peripheral Neuropathy

**DOI:** 10.3390/cells9061553

**Published:** 2020-06-26

**Authors:** Mohammed M. H. Asiri, Sjoukje Engelsman, Niels Eijkelkamp, Jo W. M. Höppener

**Affiliations:** 1 Center for Translational Immunology, University Medical Center Utrecht, Utrecht University, 3584 EA Utrecht, The Netherlands; M.M.H.Asiri@umcutrecht.nl (M.M.H.A.); s.engelsman@students.uu.nl (S.E.); J.W.M.Hoeppener@umcutrecht.nl (J.W.M.H.); 2 The National Centre for Genomic Technology, Life Science and Environment Research Institute, King Abdulaziz City for Science and Technology, P.O. Box 6086, 11461 Riyadh, Saudi Arabia; 3 Center for Molecular Medicine, University Medical Center Utrecht, Utrecht University, 3584 EA Utrecht, The Netherlands

**Keywords:** amyloid proteins, amyloidosis, type 2 diabetes mellitus, peripheral neuropathy, amyloid neuropathies, chronic pain

## Abstract

Painful peripheral neuropathy affects millions of people worldwide. Peripheral neuropathy develops in patients with various diseases, including rare familial or acquired amyloid polyneuropathies, as well as some common diseases, including type 2 diabetes mellitus and several chronic inflammatory diseases. Intriguingly, these diseases share a histopathological feature—deposits of amyloid-forming proteins in tissues. Amyloid-forming proteins may cause tissue dysregulation and damage, including damage to nerves, and may be a common cause of neuropathy in these, and potentially other, diseases. Here, we will discuss how amyloid proteins contribute to peripheral neuropathy by reviewing the current understanding of pathogenic mechanisms in known inherited and acquired (usually rare) amyloid neuropathies. In addition, we will discuss the potential role of amyloid proteins in peripheral neuropathy in some common diseases, which are not (yet) considered as amyloid neuropathies. We conclude that there are many similarities in the molecular and cell biological defects caused by aggregation of the various amyloid proteins in these different diseases and propose a common pathogenic pathway for “peripheral amyloid neuropathies”.

## 1. Introduction 

The nervous system and the endocrine system are major regulatory systems that maintain homeostasis of the body in response to both endogenous and environmental stimuli. The peripheral nervous system encompasses all neurological tissues outside of the brain and spinal cord, including 12 pairs of cranial nerves and 31 pairs of spinal nerves, along with their roots and branches [1]. Peripheral sensory neurons are vulnerable to damage because they may have very long axons and the sensory neurons in the dorsal root ganglia have an attenuated protective neurovascular barrier compared with the blood–brain barrier and blood–nerve barrier. Damage to peripheral nerves causes peripheral neuropathy [1], which can involve gain- or loss-of-function. Gain-of-function symptoms include increased pain sensitivity [2], whereas loss-of-function symptoms include hyposensitivity and even a total loss of sensation (numbness) [3,4].

Neuropathic pain affects 7–10% of the population [5]. Peripheral neuropathic pain may have multiple causes, including diabetes mellitus (DM), hereditary disorders, inflammation and infections, autoimmune diseases, kidney failure, chronic alcoholism, and certain medications—especially those used to treat cancer and HIV/AIDS [6,7]. Although various diseases or causes may be at the root of peripheral neuropathy, the precise molecular mechanisms that cause (painful) peripheral neuropathy are still not well understood [7].

Diseases caused by amyloid—extracellular deposits of aggregated, misfolded proteins—are known as amyloidoses [8,9]. Amyloidosis can be inherited or acquired. Some types of amyloidoses are life threatening, such as immunoglobulin light-chain amyloidosis (AL) and serum amyloid A protein amyloidosis (SAA) [10,11]. Intriguingly, among patients with amyloidosis, peripheral neuropathy is observed in a substantial number, and for some types of amyloidosis this occurs in all patients, pointing towards amyloid being a cause of peripheral neuropathy (see Table 1). 

**Figure 1:**
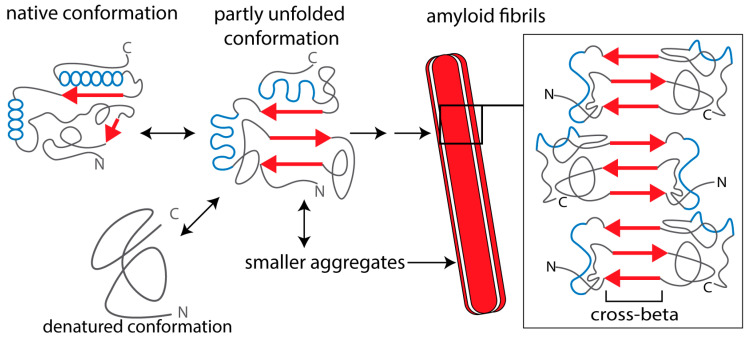
Schematic representation of amyloid protein aggregation. The β-strands in the amyloid-forming protein are indicated as red arrows and α-helices as blue spheres. In the native conformation, β-strands of the monomeric protein (if present) are not aligned and “shielded”, which prevents intermolecular aggregation. A partially unfolded or misfolded molecule can form different kinds of intermolecular aggregates. Amyloid oligomers are relatively small, compact structures that may be composed of antiparallel β-strands or contain α-helical conformations. Protofibrils and mature amyloid fibrils are formed via β-strand stacking, forming extended networks of β-sheets with a characteristic cross-beta structure. Mature fibrils consist of a few identical fibrillar “subunits”. Smaller aggregates (oligomers and protofibrils) are mostly cytotoxic, whereas extracellular, fibrillar amyloid deposits can also impair tissue and organ function by impairing blood supply to the cells (see text for references).

Amyloid is characterized by fibrils with diameters ranging 7–10 nm that are composed of stacked β-strands held together by hydrogen bonds [8,9,12]. Since the longitudinal axis of the amyloid fibrils is perpendicular to the plane of the β-strands, this is also referred to as the cross-β structure of amyloid fibrils (Figure 1) [8,10,12,13]. Amyloid deposits consist for ~90% of a fibril-forming protein, which is specific for each amyloid disease. However, all amyloid deposits contain common components that are involved in the amyloid formation process, such as serum amyloid P protein (SAP), heparan sulfate proteoglycans (HSPG), and apolipoprotein E (ApoE) [14].

Amyloid fibrils are present extracellularly and deposit locally at specific sites in the body, often the site of amyloid protein production, or systemically with involvement of multiple organs. Amyloid depositions cause cellular damage, tissue injury, and organ failure. For example, amyloid deposits impair appropriate supply of nutrition and oxygen to tissues or induce inflammatory reactions that cause tissue damage, including damage to nerves [15]. Based on these findings, the dogma was that mainly mature amyloid fibrils are responsible for cell dysfunction and cell death in amyloid diseases. However, fibril-forming proteins also form smaller aggregates, protofibrils, and oligomers (Figure 1), which are actually the main cause of the cellular toxicity of amyloid-forming proteins. Hence, these oligomers are often referred to as “toxic oligomers” [16]. 

Amyloid deposition may lead to neuropathy, as is well-established for some rare, familial amyloid neuropathies and acquired amyloid polyneuropathies [17,18,19,20,21], as well as several familial autoinflammatory syndromes [22,23]. However, for more common acquired amyloid diseases, not much is known about the development of peripheral neuropathy in these diseases. The most common disease associated with amyloid formation and peripheral neuropathy is diabetes mellitus type 2 (T2DM). Amyloid in the pancreatic islets of Langerhans is a characteristic feature of T2DM [24]. Diabetic peripheral neuropathy (DPN) is one of the most common complications of T2DM and affects nearly 50% of adults with DM during their lifetime [25]. As discussed in this review, several findings point to the amyloid protein in T2DM, islet amyloid polypeptide (IAPP), as a cause in the development of DPN. In addition, in various chronic inflammatory diseases, notably rheumatoid arthritis (RA), osteoarthritis (OA), psoriatic arthritis (PA) and inflammatory bowel diseases (IBD), both peripheral neuropathy and amyloid have been described; however, a causal relation has not yet been established.

Some indications, mainly from animal models, suggest that peripheral neuropathy may also develop in central amyloid neuropathies (notably Alzheimer’s disease, Parkinson’s disease, and prion diseases) where amyloid proteins and oligomers are also present outside the central nervous system [26,27]. However, due to their predominant central neuropathic nature, these neurodegenerative diseases will not be discussed here. 

In this review, we will discuss how amyloid can cause neuropathy and address whether a common pathogenic pathway of “peripheral amyloid neuropathy” exists in both rare, familial amyloid neuropathies and acquired amyloid neuropathies, as well as in common diseases in which peripheral neuropathy and amyloid co-occur, such as T2DM and chronic inflammatory diseases, which are not (yet) considered as amyloid neuropathies.

## 2. Causes of Amyloidogenesis 

At present, 36 proteins have been identified as amyloid fibril proteins in humans. These amyloid fibril proteins affect different tissues and are associated with diseases with highly variable prevalence [8]. The mechanisms causing aggregation or fibrillogenesis of these amyloid proteins are diverse. Various amyloidoses are characterized by the development of peripheral neuropathy. Thus, neuropathy develops in these diseases independent of the nature of the respective amyloid fibril protein and of the location of amyloid protein production [10,28] (See Table 1). 

Some amyloidogenic proteins have an inherent tendency to form amyloid fibrils due to their three-dimensional structure. Among these is transthyretin (TTR), which causes wild-type TTR amyloidosis (wtATTR; also called senile systemic amyloidosis, SSA) [29] The development of amyloid in this disease is likely promoted by age-related, post-translational modifications of the TTR protein [30].

A well-established factor that promotes amyloid formation is high protein concentration. In dialysis-related amyloidosis, β2-microglobulin (β2M) concentrations in blood are elevated more than 60-fold in end-stage kidney failure due to ineffective renal clearance [31]. Similarly, serum amyloid A protein (SAA) is produced at high quantities under inflammatory conditions [32,33]. These high blood levels of SAA are essential for amyloidosis to occur; however, specific SAA gene polymorphisms are also a risk factor [23]. The notion that high protein concentration is a risk factor for amyloidosis is further supported by the finding that plasma cell or B lymphocyte proliferative disorders may cause excessive production of immunoglobulin light chains, leading to AL amyloidosis (primary systemic amyloidosis) [34]. In T2DM, IAPP is overproduced together with insulin in pancreatic islet β cells in order to compensate for insulin resistance, which promotes formation of islet amyloid deposits [24,35,36,37]. 

Another risk factor for amyloidosis is mutation of a gene for which the non-mutated version encodes a protein that is non-fibrillogenic. Such mutations are at the root of several hereditary forms of amyloidosis [38,39]. The causative mutations either modify proteolytic cleavage of a precursor protein or alter the protein structure, resulting in proteins with intrinsic tendency to form amyloid fibrils. In familial amyloid polyneuropathies (FAP), which are associated with peripheral neuropathy, mutations in the genes encoding TTR, gelsolin, or apolipoprotein A1 cause amyloid formation [39,40]. Amyloidosis (from the SAA protein) and peripheral neuropathy also occur in some familial autoinflammatory diseases, although the primary genetic defect in these syndromes does not occur in an amyloid protein gene [22].

## 3. Peripheral Amyloid Neuropathies

**Figure 2:**
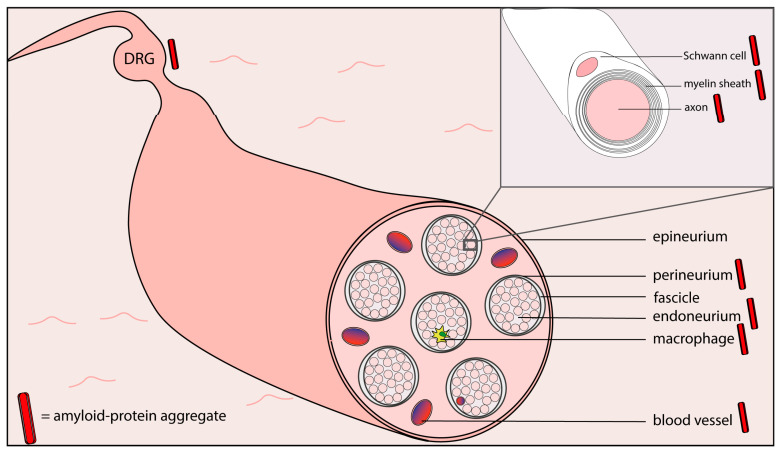
Schematic representation of the ultrastructure of a peripheral sensory nerve, with locations where amyloid or amyloid protein aggregates have been demonstrated indicated by a red bar (based on references [73,74,75,76,77,78,79,80,81]). DRG = dorsal root ganglion.

In Table 1, several characteristics of hereditary and acquired diseases with both amyloidosis and peripheral neuropathy are listed. In several of these diseases, amyloid deposits, or amyloid protein aggregates, have been found at different locations within peripheral nerves (see Figure 2). 

**Table 1:** Diseases with known peripheral nervous system involvement and a (potential) link to amyloid proteins.

Disease	Amyloid Protein	Acquired/ Hereditary	Local/ Systemic	Peripheral Nervous System Involvement	Prevalence/Incidence Disease	Prevalence Disease w/w *	Prevalence/Incidence PN (% of Patients)
**Familial amyloid polyneuropathy**	Transthyretin (hATTR)	Hereditary	Systemic	Polyneuropathy Autonomic disturbances Carpal tunnel syndrome [41]	10,186 persons w/w (range: 5526–38,468)/UN [42]	0.00013%	UN (it develops in the majority of patients/UN [42,43]
	Apolipoprotein A-I (AApoAI)	Hereditary	Systemic	Polyneuropathy [44]	UN/UN	UN	UN/UN
	Gelsolin (HGA)	Hereditary	Systemic	Cranial neuropathy Polyneuropathy [28]	400 to 1000 gene carriers in Finland/UN [45]	0.01%	UN/UN
**Immunoglobulin light-chain amyloidosis**	Ig light-chain	Acquired	Systemic	Polyneuropathy Autonomic disturbances Carpal tunnel syndrome [46,47]	40.5 cases per million in the US/9.7 to 14.0 cases per million per year in US [48]	0.004%	15–20%/UN [49]
**Dialysis-related amyloidosis**	β2-microglobulin	Acquired	Systemic	Carpal tunnel syndrome Polyneuropathy [50,51]	UN/UN (incidence > 95% in. patients > 15 years dialysis in US) [51]	UN	UN/UN
**Senile systemic amyloidosis**	Transthyretin	Acquired	Systemic	Polyneuropathy Autonomic disturbances carpal tunnel syndrome [52]	63/256 of the study population in Finland (25% > 80 years old)/UN [53]	0.45%	UN/UN
**Type 2 diabetes mellitus**	IAPP	Acquired	Local/systemic	Polyneuropathy [54]	463 million persons (aged 20–79 years) w/w (including T1DM&T2DM)/UN [55]	5.4%	31.5–45% [56,57]
**Rheumatoid arthritis**	SAA	Acquired	Systemic	Polyneuropathy [58]	19,965,115 persons w/w/1,204,599 new cases w/w [59]	0.26%	39.19–75.28% [58]
**Inflammatory bowel disease**	SAA	Acquired	Systemic	Polyneuropathy [60,61]	68 million persons w/w/70,000 new cases per year in USA [62,63]	0.09%	UN/0.07% after 10 years of IBD [60,64]
**Osteoarthritis**	TTR, Apo-A1	Acquired	Systemic	Polyneuropathy [65]	303 million persons w/w (80% of people > 75 years)/14.93 million new cases w/w [66,67]	3.9%	UN/UN
**Psoriatic Arthritis**	SAA	Acquired	Systemic	Polyneuropathy [68]	133 per 100,000 persons w/w/83 per 100,000 persons per year w/w [69]	0.133%	UN/UN
**Familial Mediterranean fever**	SAA	Hereditary	Systemic	Polyneuropathy [22]	100,000 persons in Turkey /UN (high among people from the eastern Mediterranean e.g., Arabs, Turks, Jews, and Armenians) [70,71]	0.13%	UN/UN
**Muckle–Wells syndrome**	SAA	Hereditary	Systemic	Polyneuropathy [22]	Rare, MWS is one of the three clinical forms of CAPS and the prevalence of CAPS is 1–10 cases per million in France/UN [72]	0.001% (based on max 10 per million)	UN/UN

Abbreviations: CAPS = cryopyrin-associated periodic syndrome; HGA = hereditary gelsolin amyloidosis; hATTR = transthyretin-associated hereditary amyloidosis; IAPP = islet amyloid polypeptide; IGT = impaired glucose tolerance; MWS = Muckle–Wells syndrome; PN = peripheral neuropathy; SAA = serum amyloid A protein; T1DM = type 1 diabetes mellitus; T2DM = type 2 diabetes mellitus, UN = unknown; w/w = worldwide, * The calculated numbers for estimation of the worldwide prevalence are based on the: (1) same prevalence in every country; (2) world population = 7.6 billion, worldwide population aged over 80 years = 143 million, US population = 327 million, Finnish population = 5.5 million, Turkish population = 80 million. Although the prevalence of some diseases may be very different in different parts of the world, these (albeit artificial or fictional) numbers enable a global comparison of the prevalence of these diseases.

### 3.1. Familial and Acquired Amyloid Polyneuropathies

Familial amyloid polyneuropathy (FAP) is the collective name for three inherited autosomal dominant disorders caused by germline mutations in genes encoding amyloidogenic proteins. Acquired neuropathic amyloidosis refers to systemic amyloidosis, which are associated with peripheral neuropathy but are not caused by a mutation. These diseases may develop secondary to another disease or as a consequence of aging. Among 36 amyloid fibril proteins identified in humans, only a few have been associated with acquired peripheral neuropathy [8,82].

Although the prevalence of peripheral neuropathy in the various amyloid diseases may be quite variable (see [83]) and the relevance of amyloid protein aggregation for the development of peripheral neuropathy in these disease may also vary, some common characteristics do emerge. Peripheral amyloid neuropathy usually presents as symmetric polyneuropathy, which is length-dependent, i.e., beginning in the lower extremities and extending more proximally as the disease progresses [84,85,86]. This nature of the disease, primarily affecting the longest axons, is likely due to defects in the axonal transport [87] or mitochondrial function [79,80,81,88], because both are important for maintaining the integrity of long axons. Initially, both myelinated and unmyelinated small fibers are affected, with decreasing nerve fiber density in later stages of the disease [89]. With disease progression, large-myelinated fibers are also affected and loss of motor neurons even occurs in some of these diseases [40]. In addition to peripheral sensory nerves and DRGs [74,90,91], amyloid has also been detected in autonomic nerves [92] and autonomic ganglia [21]. As a consequence, autonomic dysfunction is present in several of the amyloid diseases (Table 1 and [83]). Development of signs and symptoms of autonomic dysfunction, however, usually occurs later than the peripheral polyneuropathy [93]. 

Amyloid and oligomers have been detected several years before the beginning of neuropathy [75,94,95,96], suggesting that amyloid protein aggregation may be involved early in the pathogenesis of the neuropathy. For hereditary apolipoprotein A-I amyloidosis (AApoAI), various ApoA-I gene mutants cause (poly) neuropathy, however not all amyloidogenic apoA-I gene mutations lead to neuropathy [97], indicating a genotype–phenotype correlation. Neuropathy in hereditary gelsolin amyloidosis (HGA) might be caused by both amyloid formation and loss of function of the gelsolin protein [98], since gelsolin is a key regulator of actin filament assembly and disassembly [99,100], and in neurons these processes govern spine formation, morphology, and synaptic functions [101]. For the familial autoinflammatory syndromes, familial Mediterranean fever and Muckle–Wells syndrome, amyloidosis from the SAA protein is suggested as cause of the peripheral neuropathy, although the primary genetic defect occurs in another gene [22,23].

**Figure 3:**
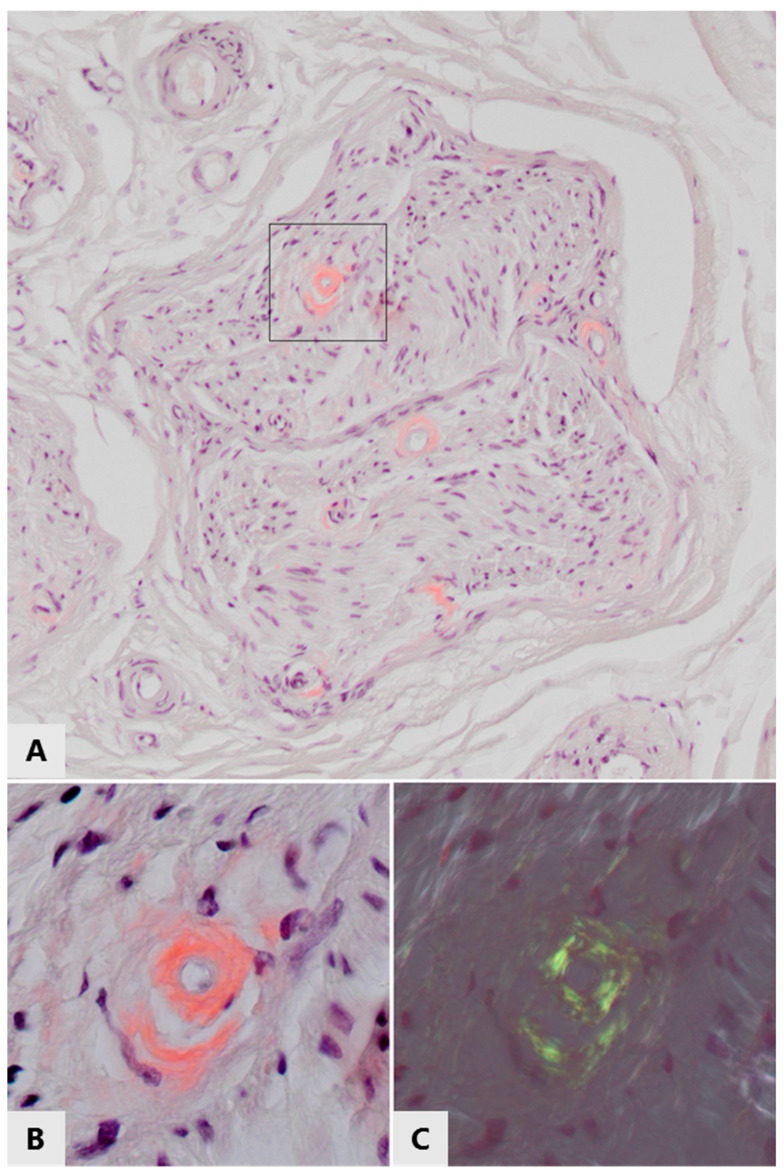
Amyloid deposits in/around small endoneurial blood vessels in the left sural nerve of a 70-year old patient with immunoglobulin light chain amyloidosis. (**A**) White light microscopy of Congo red stained section, showing pink-stained thickening of vascular walls in a nerve fasciculus. These thickened vascular walls also stained positive for lambda light chains (not shown). (**B**,**C**) Enlargement of the framed area of the top panel, (**B**) viewed with white light, (**C**) viewed with polarized light, showing green/yellow birefringence of the Congo red positive vascular walls, proving the amyloid nature of these light chain deposits.

AL amyloidosis, an acquired neuropathic amyloidosis, is caused by overproduction of antibodies in plasma cell or B lymphocyte proliferative disorders, such as multiple myeloma. Immunoglobulin light chains aggregate and form amyloid deposits, for example in nerves ([48,102,103,104,105], see Figure 3). In dialysis-related amyloidosis (DRA), β2 microglobulin gradually accumulates in blood when it cannot pass the dialysis membrane and the most frequent polyneuropathy is distal axonal sensorimotor polyneuropathy [106,107,108]. Thanks to general improvements in dialysis care, the prevalence of this type of amyloidosis appears to be decreasing [109]. In senile systemic amyloidosis (SSA), age-related deposition of wtTTR amyloid is found in several organs, while carpal tunnel syndrome is often the first clinical manifestation of the disease [29,110]. Oxidation of methionine and cysteine residues of TTR increases with age and promotes aggregation of wild-type TTR; therefore, these post-translational modifications have been implicated in the pathogenesis of SSA [30]. In contrast to hATTRv, a subtype of FAP where neuropathy is frequent, neuropathy is either not frequent or underdiagnosed in SSA [28], suggesting that mutant TTR is more neurotoxic than wtTTR.

Both in familial amyloid polyneuropathies and in acquired amyloid polyneuropathies, other organs than the nervous system can be affected, notably the liver, kidney, heart, and gastrointestinal tract [44,53,92,111,112,113,114,115,116,117]. Why these particular tissues are commonly affected is not known. 

### 3.2. Common Acquired Diseases with Peripheral Neuropathy and Amyloid

Apart from the mostly rare amyloid neuropathies described above, some common acquired diseases also are associated with peripheral neuropathy and amyloid. However, little is known about the potential contribution of the amyloidogenic protein in the pathogenesis of peripheral neuropathy in these diseases, and these diseases are not (yet) classified as peripheral amyloid neuropathies. The possible causative relation of amyloid and toxic oligomers with peripheral neuropathy in these diseases will be discussed next, with a specific focus on type 2 diabetes mellitus. 

#### 3.2.1. Type 2 Diabetes Mellitus (T2DM)

In 2019 the International Diabetes Federation (IDF) indicated that there were ~450 million people with DM worldwide. This number is expected to rise to 700 million by 2045 [55]. Type 1 DM is characterized by autoimmune-mediated loss of the insulin-producing β-cells in the pancreatic islets of Langerhans, causing insulin insufficiency and hyperglycemia [118]. T2DM is the most common type of DM, accounting for approximately 90% of all DM patients [55]. T2DM is characterized by both insulin resistance (reduced insulin sensitivity of insulin target tissues as a consequence of obesity) and β-cell failure (insulin insufficiency), leading to hyperglycemia. Increased β-cell apoptosis in T2DM is associated with glucotoxicity, lipotoxicity, and deposition of amyloid in the pancreatic islets [119,120,121]. Islet amyloid is a characteristic histopathological feature of T2DM, being detected in approximately 90% of T2DM patients at autopsy [24]. However, islet amyloid has recently also been detected in 3 young patients with T1DM [122].

Peripheral neuropathy is the most frequent chronic complication of DM. The prevalence of peripheral neuropathy in DM ranges from 10% at one year after DM diagnosis to more than 50% during progression of the disease [4,25,123,124], making diabetic peripheral neuropathy (DPN) the most abundant type of peripheral neuropathy worldwide [3,4,125] (Table 1). Diabetic neuropathy that is painful develops in approximately 50% of DM patients with neuropathy [126]. DPN is a major cause of lower limb amputation, which severely affects both quality of life and life expectancy [127]. Peripheral neuropathy in T2DM is poorly managed clinically because of its late diagnosis, complex pathogenesis, and the limited therapeutic options to treat neuropathy [4]. Long-lasting DM causes loss of sensory peripheral nerve terminals. At the early stage of DPN, small nociceptive sensory fibers are commonly affected. Motor function is hardly affected, although some slowing of motor conduction velocity is observed [128]. The symptoms of DPN involve gain- or loss-of-function, depending on the type of nerve that has been damaged. Gain-of-function symptoms include allodynia (feeling of pain from non-painful stimuli) and hyperalgesia (increased pain sensitivity), whereas loss-of-function symptoms include tactile and thermal hyposensitivity [3,4]. Patients can experience pain in some areas of the body and loss of sensitivity in other areas [3,124]. During progression of DPN, patients can even develop a total loss of sensation (numbness), which contributes to development of complications such as diabetic foot ulcers [25]. The symptoms of DPN tend to follow a “stockings and gloves” pattern, which means that they start at the feet and hands [3,124]. 

Hyperglycemia is generally considered a primary cause of DPN [3,124]. Several hyperglycemia-induced molecular pathways contribute to deregulation of neuronal function, including the polyol pathway, hexosamine pathway, activation of PKC isoforms (notably α, β1, β2, δ, and ε), and formation of advanced glycation end products (AGEs), among others [3,129,130,131,132,133]. These pathways and molecules cause microangiopathy, oxidative stress, and inflammation, which contribute to cytotoxic effects on neurons and Schwann cells, leading to nerve fiber loss and axonal degeneration, and consequently loss of sensory perception (reviewed in [130,131,132,133,134]). However, some data indicate that other factors besides hyperglycemia play a role in the development of DPN in T2DM. For example, tight blood glucose control is able to reduce hyperglycemia and diminish neuropathy in T1DM [135], but in T2DM improved glycaemia is not, or only partly, accompanied by less severe neuropathy [135,136]. Moreover, neuropathy is also present in individuals with prediabetes (i.e., not yet having developed hyperglycemia) [137], indicating factors other than hyperglycemia are involved. Large clinical studies support the concept that components of the metabolic syndrome, (notably obesity and prediabetes) which include elevated levels of the amyloidogenic protein hIAPP, may underlie the pathogenesis of DPN, especially in T2DM [138]. 

IAPP is the fibril-forming protein of pancreatic islet amyloid, which is composed of 37 amino acids and is co-produced and co-secreted with insulin from the pancreatic islet β-cells [36,139,140]. The physiological functions of IAPP are not fully understood, but include enhancement of satiety, reduction of gastric emptying and of glucagon release, and inhibition of insulin signaling [36,139]. Insulin resistance leads to increased production of insulin to compensate for its impaired signaling, which is accompanied by increased production of IAPP [35,36]. Moreover, free fatty acid levels in the blood increase with obesity, promoting IAPP gene expression and secretion by islet β-cells [141]. Elevated concentrations of human IAPP (hIAPP) trigger formation of toxic oligomers and amyloid plaques, which impair islet function and increase β-cell apoptosis [24,140,142]. In contrast to hIAPP and IAPP from monkeys and cats, murine and rat IAPP are not amyloidogenic due to differences in the amino acid sequence [143,144]. Therefore, rodent models with islet β-cell-specific expression of a hIAPP transgene have been developed to study the pathogenic role of hIAPP and islet amyloidosis [145]. In T2DM, deposits of aggregated hIAPP are found in the pancreatic islets, but also elsewhere in the body, i.e., in the heart, kidneys, and brain [146,147,148], indicating that amyloidosis in T2DM is not restricted to the pancreas. Apart from the pancreatic islet β-cells, IAPP is also expressed in peptidergic sensory neurons [149]. Several mouse studies revealed that IAPP has an excitatory role in nociception [150,151,152,153,154,155]. Therefore, with the knowledge that several other amyloid proteins cause peripheral neuropathy, we hypothesized that aggregated hIAPP causes peripheral neuropathy in individuals where hIAPP is overproduced, as is the case in (development of) T2DM. 

Injection of hIAPP in wild-type mice induces mechanical hypersensitivity and reduction in nerve fiber density [156]. More importantly, in a more physiologically relevant model system, i.e., transgenic mice endogenously expressing hIAPP specifically in pancreatic islet β cells [142,157], mechanical hypersensitivity develops and skin nerve fibers are reduced [156]. Thus, hIAPP causes signs of peripheral neuropathy in vivo, even in the absence of hyperglycemia. In further support of hIAPP as a driver of painful neuropathy, others reported that IAPP modulates neuropathic pain in mice and rats at different levels of the nervous system [154,158]. Moreover, hIAPP is involved in central neuropathy, both in Alzheimer’s disease patients with T2DM [159] and in diabetic hIAPP transgenic rats [159,160], further substantiating the neuropathic potential of aggregated hIAPP. Overall, these data support the hypothesis that hIAPP is a driver of peripheral neuropathy in T2DM, but since the clinical data are only correlative, further investigation into the role of hIAPP in peripheral neuropathy in human T2DM is warranted. 

#### 3.2.2. Acquired Chronic Inflammatory Diseases

Serum amyloid A protein amyloidosis (SAA) is a major complication of chronic inflammation and one of the most common human systemic amyloid diseases worldwide. Serum amyloid A protein is synthetized in large quantities in chronic inflammatory diseases and can lead to amyloid deposits in any chronic inflammatory disorder [161,162]. Amyloid deposits are mainly found in kidney, subcutaneous adipose tissue, and gastrointestinal mucosa. Peripheral neuropathy is not considered as a characteristic feature of this systemic amyloid disease. However, in several chronic inflammatory diseases, notably in rheumatoid arthritis, osteoarthritis, psoriatic arthritis, and to a lesser extent inflammatory bowel diseases, peripheral neuropathy is present in a subset of patients, and amyloid has been demonstrated in these diseases [58,163] (see Table 1). For example, in sural nerve biopsies of rheumatoid arthritis patients, perineurial thickening with amyloid was detected in 4 out of 23 patients investigated [163]. In knee joints of 12 out of 12 osteoarthritis patients, as well as 7 out of 12 aged individuals without osteoarthritis, amyloid was present in menisci, articular cartilage, and synovial membranes, mostly of TTR and Apo-AI origin [164,165]. In psoriatic arthritis, amyloid has been reported in 30 cases and was present mainly in the kidney [166]. In Crohn’s disease, SAA-protein-derived amyloid is found in the kidney and gastrointestinal tract in 0.3–10% of patients [167,168,169]. 

Whether there is a causal relation between the development of peripheral neuropathy and amyloid in these diseases is not known, because literature on peripheral nervous system involvement in inflammation-related amyloidosis is scarce. However, three cases reports of patients with peripheral neuropathy and SAA protein amyloid exclusively within axons and myelin sheaths underscore a potential link between SAA amyloid and peripheral neuropathy [78]. Thus, amyloid protein aggregation might also be involved in development of peripheral neuropathy in chronic inflammatory diseases. 

The presence of amyloid in histological specimens is routinely demonstrated by Congo red staining of tissue sections and subsequent yellow-green birefringence when viewed with polarized light (see Figure 3). The reported prevalence of amyloid detected with this method within the peripheral nervous system in T2DM and chronic inflammatory diseases is low, or has even not been investigated or reported at all. However, this technique only detects fibrillary amyloid deposits, not prefibrillar aggregates such as oligomers. Notably, these oligomers are generally thought to be the most cytotoxic species of amyloid protein aggregates. In addition, amyloid deposits are generally larger, and thus more readily detectable in organs such as the kidney and heart as compared to nerves or even individual neurons. We propose that pathogenic involvement of amyloid protein aggregation in peripheral neuropathy may be underestimated in some diseases not (yet) considered as amyloid neuropathies. 

As peripheral sensory neuropathy is a hallmark of several amyloidoses, T2DM, and some inflammatory diseases, the question arises as to why sensory neuropathy is most prominent in these diseases. Damage to peripheral sensory nerves may be noticed or diagnosed sooner as compared to other nerves. Nevertheless, peripheral neurons, in particular long axons, may be particularly sensitive to degeneration and to toxic compounds due to their effects on key processes essential to maintaining homeostasis in these long axons, such as protein transport, membrane integrity, and mitochondrial function. Some sensory neurons are not covered by myelin sheets, which may render them even more susceptible. Finally, sensory neurons may have a particular sensitivity towards oligomers because of the composition of plasma membrane lipids, which might promote oligomer toxicity. Although these aspects may contribute to the observed presence of neuropathy in amyloidosis, future research will have to address these issues.

To further support potential links between amyloid or toxic oligomers and the development of peripheral neuropathy in these diseases, we will discuss the mechanisms as to how amyloid and amyloid protein aggregates may contribute to peripheral neuropathy. 

## 4. Mechanisms Linking Amyloid and Peripheral Neuropathy

**Figure 4:**
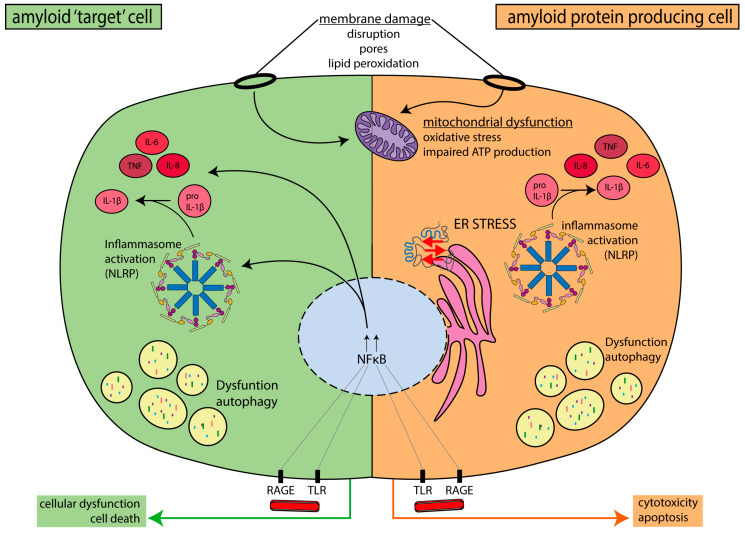
Overview of the cell biological mechanisms that have been implicated in amyloid-protein-induced cellular damage and apoptosis and peripheral neuropathy, both in cells producing an amyloid protein and in other cells affected by amyloid or amyloid protein aggregates. Such “amyloid target cells” include cell types that degrade amyloid and protein aggregates after phagocytosis (macrophages and microglia), as well as Schwann cells (involved in nerve function and integrity) and endothelial cells (involved in microangiopathy).

Amyloid and toxic oligomers may cause cell damage through various mechanisms, including membrane disruption, impaired mitochondrial function, autophagy dysfunction, and others. Many of these cellular deficits are involved in the development of peripheral neuropathy. These deficits may occur not only in amyloid-protein-producing cell types, such as hepatocytes, pancreatic islet β-cells, and neurons, but also in other cell types involved in the development of neuropathy, such as macrophages or microglia, Schwann cells, and endothelial cells. The information described below on the cell biological mechanisms in the pathogenesis of amyloid toxicity, including (peripheral) neuropathy, is summarized in Figure 4.

### 4.1. Protein–Membrane Interactions

Interaction of amyloid proteins with membrane phospholipids promotes fibril formation of SAA [170,171,172], Aβ [173,174], hIAPP [175,176], and apolipoprotein C-II [177]. In vitro, hIAPP amyloid fibril formation occurs at or in the cell membrane [175]. When interacting with membranes, amyloid proteins may adopt α-helical conformations that further promote aggregation [178,179]. Membranes are also a target for cytotoxic actions of amyloid protein aggregates, as is extensively demonstrated for SAA [180], hIAPP, Aβ, and α-synuclein [181,182,183]. Amyloid proteins disrupt the integrity of the phospholipid bilayer, but oligomers also form small pores in the plasma membrane that can act as non-specific ion channels, called “amyloid pores” [184,185]. In neurons, such pores could lead to membrane depolarization, causing ectopic discharges and neuronal damage. Basal membranes and organelle membranes (e.g., of the mitochondria, endoplasmic reticulum, and nucleus) are also affected by amyloid protein aggregates [186,187,188]. Amyloid-mediated disruption of membrane integrity increases the exposure of polyunsaturated fatty acids to cytosolic reactive oxygen species (ROS) leading to formation of reactive aldehydes. For example, Aβ causes hydroxyl radical generation, which induces membrane lipid peroxidation through hydrogen abstraction from polyunsaturated fatty acids [189]. Generated reactive aldehydes further elevate ROS production and trigger inflammatory responses [190]. Human IAPP and Aβ cause membrane lipid peroxidation in primary neurons, which triggers calcium influx and IL-lβ synthesis [159,191]. Inhibitors of lipid peroxidation reduced mechanical hypersensitivity in a rat model of neuropathic pain [192], supporting a role of lipid peroxidation in the development of peripheral neuropathy.

### 4.2. Endoplasmic Reticulum (ER) Stress

The endoplasmic reticulum (ER) is the main cellular compartment involved in protein folding and secretion. Disturbances in ER homeostasis (e.g., due to aberrant or misfolded proteins) induce ER stress. ER stress triggers the activation of the unfolded protein response (UPR), a response which alters the expression of genes involved in ER quality control. The UPR aims to recover ER homeostasis or trigger apoptosis when cells are irreversibly damaged. As cells age, their ability to maintain a balance in protein folding or degradation and to cope with disruptions in proteostasis declines. This decline contributes to the development of age-related diseases, including neurodegenerative diseases [193,194] and putatively acquired amyloidosis, along with its complications. 

Amyloidogenic proteins, either wild-type or mutant, have a tendency to misfold and aggregate, which puts pressure on the ER, particularly in cases of overproduction (e.g., SAA in inflammation and hIAPP in insulin resistance and T2DM) or production of a mutant protein with acquired amyloidogenic potential. Thus, it is not surprising that ER stress is a major mechanism implicated in cytotoxicity in amyloidoses. Increasing evidence indicates that the toxic molecular species that evoke ER stress are the intermediate oligomeric forms and not the mature amyloid fibrils [195]. 

In systemic amyloidoses, the amyloidogenic proteins evade ER quality control, causing progressive aggregation and amyloid deposition. Regulation of ER quality control, therefore, is a crucial mechanism in defining the onset and progression of systemic amyloid diseases [196]. TTR aggregates and ER stress are present in dorsal root ganglia of TTR transgenic mice [197], suggesting a role in familial amyloid polyneuropathy. 

For hIAPP and Aβ, there is extensive evidence that these proteins cause ER stress in cell and mouse models of T2DM [198,199,200,201,202,203,204] and AD [205,206,207], respectively. ER stress is also a determining factor for the onset and progression of diabetic peripheral neuropathy (DPN) [208,209]. ER stress is involved in DPN in rodent models of T2DM [209]. For example, in diabetic rats, sensory neurons display ER stress, and inhibitors of ER stress reduce the development of neuropathic pain in these animals [210]. Moreover, reduction in ER stress with a chemical chaperone in diabetic rats reduces DPN, despite continuous hyperglycemia. Thus, ER stress independent of hyperglycemia promotes peripheral nerve damage [211]. Conversely, induction of ER stress in healthy animals generates an immediate and lasting painful phenotype that is reversible by ER stress blockers [210]. Finally, ER stress is involved in other forms of peripheral neuropathy (see [212]), pointing to a central role of ER stress in peripheral neuropathy in general. 

### 4.3. Mitochondrial Dysfunction 

Neurons are cells with a complex morphology, long lifespan, and high energetic requirements. Mitochondria are essential energy suppliers that maintain the viability and proper function of neurons. Mitochondria generate ATP, buffer cytoplasmic calcium (Ca^2+^) levels, and are a source of metabolites required for neuronal functioning [213,214,215]. Thus, neurons are highly dependent on adequate mitochondrial function to maintain neuronal integrity and activity [79,80,81]. Because of the unique length and energy requirements of peripheral nerves, appropriate mitochondrial function and distribution along nerves is of fundamental importance. In general, mitochondrial dysfunction, which may lead to oxidative stress, is a main cause of axonal injury in peripheral neuropathy. Mutations in genes involved in mitochondrial fusion, fission, and axonal transport are linked to inherited peripheral nerve diseases, with demyelination and axonal defects as a consequence [88]. Moreover, peripheral neuropathy is associated with mitochondrial dysfunction, such as reduced oxidative phosphorylation, reduced ATP production, increased production of reactive oxygen species (ROS), and altered mitochondrial transport (reviewed in [216,217]). 

For amyloid neuropathy, some genetic evidence supports mitochondrial involvement. Polymorphisms in mitochondrial genes are associated with early onset of disease, i.e., with a progressive sensorimotor and autonomic axonal polyneuropathy [218], and with a sex-specific effect on penetrance of the disease in FAP patients or families [219]. 

Although experimental proof for mitochondrial dysfunction in peripheral amyloid neuropathy is lacking, overwhelming evidence exists that impaired mitochondrial function is a causative factor in T2DM (reviewed in [220]) and in the central amyloid neuropathies, such as AD and Parkinson’s disease [187,221,222,223]. Common cellular defects caused by hIAPP and Aβ include reduced activity of mitochondrial respiration and increased ROS generation [224]. Accumulation of hIAPP oligomers on mitochondrial membranes causes mitochondrial damage and β-cell toxicity, whereas an IAPP-specific ligand that binds IAPP and rescues β-cells from hIAPP-induced cytotoxicity [179] prevents mitochondrial membrane pores and toxicity [225]. Aβ oligomers downregulate mitochondrial oxidative phosphorylation [226], decrease mitochondrial potential, and increase ROS generation in rat neurons [227]. Oligomers of α-synuclein reduce axonal mitochondrial transport, disrupt axonal integrity, and reduce ATP levels in human neurons. These defects were restored by inhibition of oligomer formation [228]. 

Thus, oligomers of amyloidogenic proteins cause mitochondrial defects that play an important role in T2DM and in several neurodegenerative diseases, and likely also in amyloid-induced peripheral neuropathies. 

### 4.4. Inflammation 

Inflammation is a consequence and a cause of amyloidosis and amyloid neuropathy. In hereditary amyloidosis, inflammation results from the amyloidosis and contributes to development of the associated pathology, whereas in SAA amyloidosis, chronic inflammatory diseases are the cause for amyloid formation.

For familial amyloid neuropathies, most evidence that amyloid triggers inflammatory processes and neuropathy comes from hTTRv. In FAP/TTR, plasma levels of prototypic inflammatory cytokines are increased. Interestingly, IL-1β, IL-10, and IL-33 levels were increased already before any presence of amyloid fibril deposition in one study [229], indicating that prefibrillar aggregates caused the increase in inflammatory mediators. Early in FAP development, non-fibrillar TTR aggregates are detected in nerve biopsies that are associated with signs of inflammation, e.g., increased expression of the proinflammatory cytokines TNF and IL-1β and iNOS in nerves [90]. Toxic protein aggregates cause inflammation through activating the receptor for advanced glycation end products (RAGE) and Toll-like receptors (TLR) [230,231], which activate the transcription factor NFκB, resulting in proinflammatory cytokine gene expression [232,233]. For example, in vitro, TTR binds RAGE and activates NFκB in transfected PC-12 cells [234]. TTR fibrils also induce cytokine and iNOS expression by Schwann cells and endothelial cells in vitro in a RAGE-dependent manner [75]. Evidence implicates RAGE signalling in the pathogenesis of neuropathic pain. RAGE causes Wallerian degeneration via modulation of the inflammatory response [235], and causes sensory neuron damage in vitro by the activation of NF-κB, JAK-STAT, and ERK pathways [236]. Moreover, pharmacological inhibition of RAGE effectively attenuates development of chronic inflammatory and neuropathic pain [237]. In general, in models of neuropathic pain, inflammatory responses, including cytokine responses such as increased IL-1β and TNF, are thought to contribute to the neuropathic pain [238,239,240,241,242].

In the more common amyloid diseases with neuropathy, similar inflammatory reactions are observed as in FAP. The pyrin domain containing NOD-like receptor (NLRP3) inflammasome is a key component of the innate immune system that induces proinflammatory cytokine production. Protein aggregates from hIAPP and Aβ activate NLRP3 [243]. Similarly, aggregated SAA induces NLRP3 activation and IL-1β release in human neutrophils and keratinocytes of psoriasis patients with SAA amyloidosis [244,245]. SAA also triggers inflammatory responses through activation of TLR2, inducing IL-6 and IL-8 secretion in fibroblasts [246]. In T2DM and in obesity, pancreatic islet inflammation is implicated in β-cell dysfunction [247,248]. Extracellularly located hIAPP oligomers (but not fibrils or monomers) bind and activate RAGE, causing islet β-cell inflammation and apoptosis, whereas genetic or pharmacological inhibition of RAGE prevents hIAPP-induced β-cell apoptosis and islet inflammation [249]. Whether hIAPP-induced inflammation is the main cause for human DPN in T2DM is not yet known, but hIAPP clearly induces inflammation [249,250,251,252] and neuropathic pain [156]. In addition, accumulation of hIAPP in the brains of transgenic rats and injection of aggregated hIAPP in mice are linked to neuroinflammation and neurologic deficits [159]. Finally, serum TNF levels are higher in T2DM patients with peripheral neuropathy as compared to T2DM patients without peripheral neuropathy, providing further support for an inflammatory component in development of peripheral neuropathy in T2DM [253]. 

Additionally, local neuroinflammation is thought to be a consequence of protein aggregation in AD [254]. Aβ-induced neuroinflammation is mediated by the neuronal NLRP1 inflammasome [255], RAGE, [253,254,255,256,257] and TLR4 [256,257] in neurons. Compelling evidence has demonstrated the contribution of neuroinflammation to the pathogenesis of AD (reviewed in [258,259,260]). 

Apart from amyloid-protein-producing neurons and amyloid-clearing macrophages and microglia (discussed next), other cell types in peripheral nerves can also contribute to proinflammatory cytokine production in peripheral neuropathy.

Schwann cells express RAGE and several TLRs (with upregulation of TLR-1 in injured nerves) and inflammatory cytokine expression of Schwann cells (IL-1β, IL-6 and TNF) is upregulated following peripheral nerve injury [261]. Endothelial cells express RAGE, TLRs, NLRP-1, and NLRP-3. Activation of these receptors in response to stimuli from the bloodstream, including pathogens and damage signals, can trigger production of proinflammatory cytokines, such as IL-1β, IL-6, IL-18, and TNF [262,263,264]. Thus, both endogenously produced amyloid proteins and extracellular amyloid protein aggregates promote cytokine production by several cell types, contributing to an inflammatory phenotype that promotes apoptosis in peripheral amyloid neuropathy.

### 4.5. Macrophages and Microglia

Macrophages are central to the induction of inflammatory responses by amyloid or protein aggregates. Amyloid strongly activates macrophages to clear protein aggregates, but also causes inflammatory responses and cell death due to the inability of macrophages to process amyloid effectively. Overall, these processes may lead to neurotoxicity. For example, macrophages in the sural nerve of AL patients are activated by amyloid and phagocytose and degrade amyloid protein aggregates and fibrils [73]. Similarly, macrophages are engaged in removal of TTR deposits in FAP patients [240] and FAP mouse models [265,266,267]. Macrophages also phagocytose β2M amyloid fibrils. However, lysosomal processing of β2M fibrils by macrophages is impaired as compared to degradation of non-fibrillary SAP protein [268]. This “frustrated phagocytosis” of amyloid fibrils may contribute to death of macrophages, because endocytosed β2M amyloid fibrils cause disruption of endosomal and lysosomal membranes [269]. As a consequence of this inability of macrophages to process amyloid deposits or amyloid fibrils effectively, tissue macrophage numbers decline, which may lead to further amyloid accumulation and consequent progression of the neuropathy. Indeed, reduced numbers of tissue macrophages as compared to non-diseased control tissue were observed in amyloid-containing hearts of FAP patients [270]. Moreover, in a mouse model expressing the V30M TTR mutant, impairing macrophage recruitment increased amyloid load and expression of apoptotic cell markers [267]. These data indicate that macrophage insufficiency may lead to amyloid accumulation and disease progression in FAP patients. 

Beside causing cell death of macrophages, amyloid proteins directly activate macrophages to produce cytokines. SAA stimulates Il-1β expression in mouse and human macrophages [271,272,273]. In T2DM, macrophages serve a dual role; in early stages of hIAPP aggregation they phagocytose and degrade hIAPP aggregates and dead β-cells, while by producing IL-1β they impair islet function [252]. Mechanistically, hIAPP aggregates stimulate resident islet macrophages via TLR signalling and NLRP3 inflammasome activation to produce IL-1β, which causes β–cell apoptosis [250,251,252,274]. In support of this notion, clinical trials aimed at inhibiting IL-1β in T2DM patients showed improved β-cell function [247]. With regard to DPN, diabetes-induced neuropathy is associated with infiltration of blood-derived macrophages in the spinal cord [275], a proinflammatory phenotype of blood monocyte-derived macrophages [276] and activated spinal microglia [277]. 

Not surprisingly, amyloid proteins also activate microglia, the macrophages of the CNS. Microglia stimulate clearance of amyloid plaques, but amyloid-activated microglia release inflammatory cytokines, chemokines, and free radicals that may damage neurons. In vitro, a mutant form of TTR (A25T) activates microglia to secrete TNF, IL-6, and nitric oxide, and exposure of neuronal cultures to media conditioned by fibril-activated microglia causes synapse loss and ultimately extensive neuronal apoptosis, whilst A25T TTR fibrils are not directly toxic to neurons [278]. In vivo, intracerebroventricular injection of A25T TTR fibrils in mice induces microgliosis, increases brain TNF and IL-6 levels, and induces cognitive deficits that are prevented by the microglia inhibitor minocycline [278]. Other amyloid proteins also engage microglia. SAA stimulates IL-1β expression in rodent microglia [272,273,279]. Similar to macrophages, microglia activation by aggregated Aβ- and α-synuclein involves RAGE, TLR, and NLRP3 [280,281,282,283]. Importantly, nervous tissue infiltration or proliferation of resident macrophages and activated spinal microglia are key for initiation and maintenance of neuropathic pain [284,285], including painful diabetic neuropathy [286,287]. Thus, the presence of amyloid in nervous tissue could either aggravate macrophage and microglia responses or even initiate macrophage and microglia accumulation, promoting neuropathic pain in amyloid disease.

### 4.6. Autophagy Impairment

Autophagy is responsible for the bulk degradation of misfolded protein aggregates and damaged organelles through the lysosomal machinery. The proteasome and autophagy–lysosomal pathways are the two major routes for intracellular clearance of mutant or misfolded proteins, and protein degradation is a key cellular mechanism for protein homeostasis and cell survival. Both for cells endogenously producing amyloid proteins and macrophages and microglia removing amyloid deposits, degradation of aggregated proteins involves the autophagy–lysosomal route and not the proteasome pathway, because the narrow proteasome barrel precludes entry of oligomers and aggregates. 

The FAP TTR Y114C mutant impaired autophagy in an in vitro cell model, whereas recovery of autophagy with curcumin decreased intracellular amounts of monomeric TTR [288]. Similarly, TTR V30M aggregates caused autophagy impairment in vivo in the gastrointestinal tract of transgenic mice, which could be reversed with autophagy inducers, which also reduced apoptosis [289]. These data suggest that impairment of autophagy is linked to the pathogenesis of TTR FAP. Although experimental proof for a role in the peripheral neuropathy in FAP is lacking, autophagy dysfunction is emerging as a common pathogenic mechanism in neuropathic pain [290,291]. Notably, a variety of genes mutated in inherited peripheral neuropathies are directly or indirectly associated with the autophagy pathway [292]. 

Efficient autophagy is key to clearing hIAPP oligomers, because in hIAPP transgenic mice hIAPP oligomers were more abundant in autophagy-deficient β-cells [293,294]. Similarly, pharmacological inhibition of autophagy in vitro promotes accumulation of IAPP aggregates and death of monkey islet cells expressing amyloidogenic simian IAPP (sIAPP), but not of rodent islet cells expressing non-amyloidogenic IAPP [293]. While autophagy insufficiency leads to accumulation of hIAPP oligomers and islet cell death, conversely hIAPP oligomers impair autophagy, as evidenced by accumulation of p62, a well-known autophagy substrate [295]. This feedback mechanism may lead to a profound decrease in autophagy and a large accumulation of hIAPP oligomers or islet amyloid. Thus, autophagy–lysosomal degradation defends β cells against proteotoxicity induced by oligomerization-prone human IAPP, and as such hIAPP-induced autophagy impairment may facilitate β-cell dysfunction in the development of T2DM (also see [296]). Oligomers and amyloid also affect autophagy in neurons. Aβ accumulation in neurons causes autophagy and mitophagy abnormalities, leading to neuronal dysfunction [297,298]. In the AD brain, defects in the autophagic–lysosomal pathway and accumulation of autophagic vacuoles in dystrophic neurites are observed [299], indicating Aβ-induced dysfunctional autophagy [300,301]. 

Dysregulation of autophagy in amyloid clearing cells affects not only the phagocytosis and degradation of amyloid and protein aggregates, but also the degradation of other autophagy substrates, such as damaged organelles, dead cells, synaptic material, and myelin debris. This reduced autophagy flux promotes an inflammatory phenotype of macrophages, microglia, and apoptosis [302]. Similarly, in amyloid-protein-producing cells, including neurons [255,256], impaired phagolysosomal degradation causes proinflammatory cytokine gene expression. These inflammatory mediators, notably IL-1β and IL-6, cause cell dysfunction, pyroptosis (inflammatory caspase-mediated cell death [303]), and tissue damage [304,305]. 

In conclusion, autophagy impairment by amyloid or oligomers is well documented in both neurodegenerative and metabolic amyloid diseases, and is even considered as a treatment option for these diseases [306]. For peripheral amyloid neuropathies, evidence of a role of autophagy impairment is still limited (only shown for TTR mutants) and should be further investigated. 

### 4.7. Schwann Cells

A phenomenon that is often observed in familial amyloid neuropathy is defective myelination [91,307,308]. As such, impairment in Schwann cell function is emerging as an important mechanism in the development of amyloid neuropathy or neuropathic pain in general [309]. Schwann cells support the structural and functional integrity of peripheral nerves and are affected by the cellular toxicity of amyloid protein aggregates [75,90,310]. Schwann cells in contact with TTR amyloid fibrils become atrophic and distorted due to damage to their membranes [96,307]. Schwann cells also produce TTR, and in human TTRv transgenic mice TTR aggregates are present in the cytoplasm of Schwann cells. In addition, conditioned medium of these TTRv Schwann cells inhibited sensory neuron neurite outgrowth [311]. Thus, TTR gene expression in Schwann cells might contribute to neurodegeneration in FAP [91]. Schwann cells also produce SAA, the expression of which is upregulated by IL-6 and corticosteroids [312]. Therefore, SAA aggregates may damage Schwann cells when SAA is overproduced secondary to inflammatory diseases. 

### 4.8. Microangiopathy

Amyloid protein aggregation impairs cell function through (dys)regulation of various intracellular processes, as already discussed. However, amyloid deposits may also affect cell function and cell survival by impairment of blood flow (microangiopathy), causing reduced supply of nutrients and oxygen. In hTTRv FAP patients, amyloid fibrils are present in extracellular spaces of the endoneurium, whereas amorphous deposits are present around micro vessels and in the subperineural space [313]. It has also been described that TTRv amyloid deposits cluster around endoneurial blood vessels and even invade their walls, with subsequent occlusion of endoneurial vessels themselves [307]. TTR aggregates also induce apoptosis of endothelial cells [75]. The TTR-induced endothelial cell damage and direct compression of blood vessels by amyloid deposits likely contribute to microangiopathy in FAP [313,314]. In hereditary gelsolin amyloidosis [76] and in AL amyloidosis [113,114,115], amyloid deposits are present in and around vascular walls of nerves. Similarly, in carpal tunnel amyloidosis in dialysis patients, β2M amyloid fibrils cause endothelial basement membrane enlargement and disruption and proliferation of endothelial cells [315].

In common amyloid diseases, amyloid also causes microangiopathy, which may contribute to neuropathy. SAA affects both aortic endothelial cell and vascular smooth muscle cell function [316,317] through activation of RAGE [316] o TLR2 [318]. Pharmacological inhibition of RAGE and TLR2/4 reduces human endothelial cell dysfunction [319]. In a mouse model of SAA amyloidosis, amyloid fibril accumulation associates with lesions in basal membranes; membranes of the endoplasmic reticulum; mitochondria; and the nucleus in endothelial cells in the kidney, liver, and spleen [315]. 

In both T2DM and AD, hIAPP amyloid or aggregates are found in and around blood vessels; however, until now no reports exist on hIAPP aggregates in the vasculature of the peripheral nervous system. Nevertheless, microangiopathy is a characteristic early feature of pancreatic islet pathology in human T2DM [320], which was also the case in a HIP rat model of T2DM, where hIAPP was overexpressed [320,321]. In agreement with this notion, amyloid deposition in the pancreatic islets of humans, monkeys, and cats begins in close proximity to islet capillaries [322]. 

In T2DM patients with dementia, hIAPP depositions are present in the brain microvasculature and cause endothelial dysfunction and vessel wall disruption. Similarly, diabetic HIP rats and rats injected intravenously with aggregated hIAPP develop endothelial dysfunction and vessel wall disruption associated with neurological deficits [323]. Such endothelial disruption may lead to increased vascular permeability. Indeed, patients with diabetic polyneuropathy show increased permeability of the blood–nerve barrier [324]. This increased permeability may promote the development of neuropathy due to various mechanisms, including endoneurial edema and endoneurial osmotic imbalance [325,326]. Overall, these data indicate that hIAPP-mediated angiopathy is present in the CNS and in the pancreatic islets in T2DM. Since hIAPP-related microangiopathy is also observed outside the location of hIAPP production and because hIAPP aggregates are not restricted to this location, hIAPP might also cause angiopathy within the PNS, facilitating the development of neuropathy.

Amyloid proteins might also impair blood flow indirectly by causing damage to the autonomic nervous system, impairing vascular regulation. In T2DM, autonomic dysfunction is observed and the Aβ protein impairs sympathetic innervation of multiple organs [327].

## 5. Summary and Conclusions

Aggregation of amyloid-fibril-forming proteins causes tissue dysregulation and damage by affecting different cell types, including amyloid-protein-producing cells, amyloid-clearing cells, vascular cells, and Schwann cells. The neurotoxic actions of amyloid and the dysregulation of these other cell types may contribute to an environment that promotes damage to neurons. Apparently, similar molecular and cell biological defects are caused by aggregation of the various amyloid proteins in known (usually rare) amyloid neuropathies and in some common diseases with amyloid and peripheral neuropathy, which are not (yet) considered as amyloid neuropathies. Intriguingly similar molecular and cell biological defects are also observed in models of neuropathy. Therefore, we propose that amyloid proteins are a common cause of peripheral neuropathy in these, and potentially other, diseases. Based on the current knowledge of pathogenic mechanisms involved in amyloid-protein-induced cell and tissue damage in various amyloidoses, we propose a “model” where these mechanisms are also at the root of “peripheral amyloid neuropathies”. Since experimental proof for the applicability of this model in common amyloid diseases with peripheral neuropathy is still scarce, more research into this field is required; even more so if one realizes that neuropathic pain affects 7–10% of the global population and that underlying mechanisms in the various types of painful peripheral neuropathy are still far from understood. Therefore, understanding if and how amyloid proteins contribute to peripheral neuropathy, particularly in common acquired diseases, is of high clinical and societal relevance and may open up new strategies to prevent or treat neuropathy in these diseases
